# Implementing cardiometabolic health checks in general practice: a qualitative process evaluation

**DOI:** 10.1186/1471-2296-15-132

**Published:** 2014-07-06

**Authors:** Merijn Godefrooij, Mark Spigt, Wim van der Minne, Georgette Jurrissen, Geert-Jan Dinant, André Knottnerus

**Affiliations:** 1 CAPHRI: School for Public Health and Primary Care, Department of General Practice, Faculty of Health, Medicine and Life Sciences, Maastricht University, PO Box 616, Maastricht 6200MD, The Netherlands; 2 Woensel Primary Healthcare Centre, Eindhoven Corporation of Primary Healthcare Centres, PO Box 8736, Eindhoven 5605LS, The Netherlands

**Keywords:** Primary health care, General practice, Cardiovascular disease, Diabetes, Kidney disease, Mass screening

## Abstract

**Background:**

A stepwise screening approach for the detection and management of cardiometabolic disease is proposed in various primary care guidelines. The aim of this study was to explore the implementation of a cardiometabolic health check as perceived by the involved caregivers and patients.

**Methods:**

Qualitative process evaluation of the implementation of a cardiometabolic screening programme in a multidisciplinary primary healthcare centre in Eindhoven, the Netherlands, in which 1270 patients had participated. We explored the caregivers’ experiences though focus group discussions and collected patients’ experiences through a written questionnaire containing two open-ended questions. We analyzed our data using a thematic content analysis based on grounded theory principles.

**Results:**

Five general practitioners, three practice nurses and five medical receptionists participated in the focus groups. Additionally we collected experiences of 657 (52% of 1270) participating patients through an open-ended questionnaire.

GPs were enthusiastic about offering a health check and preferred systematic screening over case-finding, both in terms of yield and workload. The level of patient participation was high and most participants were enthusiastic about the health check being offered by their GP. Despite their enthusiasm, the GPs realized that they lacked experience in the design and implementation of a structured, large-scale prevention programme. This resulted in suboptimal instruction of the involved practice nurses and medical receptionists. The recruitment strategy was unnecessarily aggressive. There were shortcomings in communicating the outcomes of the health check to the patients and there was no predefined follow-up programme. Based on our findings we developed a checklist that can be used by designers of similar health checks.

**Conclusions:**

A number of fundamental issues may arise when GPs organize a systematic screening programme in their practice. These issues are related to the preparation of the involved staff, the importance of integration with everyday clinical practice, the approach of healthy patients and the provision of adequate follow-up programmes. The identified challenges and recommendations can be taken into account during future screening programmes.

## Background

Cardiometabolic diseases (CMD), comprising coronary heart disease, stroke, diabetes mellitus and kidney failure, are amongst the leading causes of death worldwide [1]. Awareness of the massive impact of CMD in the forthcoming years has led to a paradigm-shift from a strictly curative to a more preventive kind of medical thinking. The identification and targeting of individuals with an elevated risk for developing disease is a frequently recommended approach to primary and secondary prevention of CMD [2]. This recommendation has been adopted in various national guidelines [3-5]. A stepwise screening approach to identify high-risk individuals was recently introduced by the College of General Practitioners in the Netherlands [6,7].

Even though there is a favourable trend towards the introduction of health checks in primary care, health checks are still a matter of debate. The effects of population-wide health checks have only been demonstrated on surrogate outcomes in high-risk groups. Thus far no effects on all-cause mortality have been demonstrated [8]. Moreover, health checks lead to an in increase in new diagnoses, and may therefore lead to (over-)medicalization and increase of overall healthcare expenditure.

The evidence used to support policy decisions on whether or not to introduce these kind of screening programmes often focuses on terms such as clinical outcomes, cost-effectiveness and mortality rates [9,10]. Process evaluations of relevant studies mainly address response rates, patient uptake, follow-up and clinicians’ adherence to guidelines [11]. However, successful implementation of these evidence-based guidelines in real-life often depends on entirely different factors, such as the individually perceived value of the intervention, understanding of the intervention, time constraints, workload, clinical situations, and the motivation of both clinician and patient [12,13].

We evaluated the experiences of primary care professionals and patients during the implementation of a primary prevention programme for CMD in a medium-sized primary healthcare centre. Our main research question was: how was the process of implementing a cardiometabolic health check perceived by the stakeholders that were involved? We were also interested in the practical lessons that we could learn from these experiences.

## Methods

### Research design

We qualitatively explored experiences of primary care professionals and patients through a combination of focus group interviews and open-ended questionnaires.

### Background and setting

From mid 2008 until mid 2009 a primary prevention programme for CMD was set-up and carried out by primary care professionals from the Woensel Healthcare Centre: a multidisciplinary primary healthcare centre that is associated with the Eindhoven Corporation of Primary Healthcare Centres in the city of Eindhoven, the Netherlands. The Woensel Healthcare Centre provides a wide range of primary care (e.g. general practice, physiotherapy, social work, nutritional advice, psychology and addiction care) for approximately 7400 patients. The centre consists of six general practices, five of which participated in the CMD prevention programme.

The CMD prevention programme was a stepwise health check. All healthy patients between the ages of 40 and 75 (N = 1704) that were registered in the participating practices within the healthcare centre received a written invitation to participate in the health check by returning a short questionnaire about their current health status (age, height, weight, smoking status, physical exercise, family history of CMD and history of gestational diabetes). The questionnaire was returned by 1270 patients (75%). Based on the provided answers, those with a potentially elevated risk (n = 952, 75% of 1270) were invited for additional physical examination and lab tests. A cardiovascular risk-score was calculated and blood and urine were checked for signs of diabetes and renal failure. In 145 patients (11% of 1270) we discovered an elevated risk or manifest disease which needed further diagnostics or treatment. All participating patients received tailor-made lifestyle advice and further treatment was started when this was deemed necessary.

The CMD prevention programme was carried out by medical receptionists and practice nurses under the supervision of the participating general practitioners. The medical receptionists were responsible for sending the invitations for the health check, for calculating a simple risk score based on the answers to the returned questionnaires, for scheduling appointments for the health check, for taking a simple medical history and some physical measurements and for answering patients’ questions about the health check. The practice nurses were responsible for interpreting the outcomes of the health check, for communicating these outcomes to the patients, for providing lifestyle advice and for referring patients to a GP when necessary. More detailed procedures and outcomes of the CMD prevention programme have been published elsewhere [14].

### Primary care professionals’ experiences

We explored the experiences of primary care professionals during focus group discussions. Since three different groups of care professionals (medical receptionists, practice nurses and general practitioners) had played distinctively different roles in organizing and executing the health checks, we conducted a separate meeting for each of these groups so that every participant would feel free to openly express their thoughts and feelings. All primary care professionals that had participated in the project were invited to participate in a focus group session by the manager of the healthcare centre. The general topics that were discussed in all focus groups were:

• Overall experiences during the health check

• Experiences in working with patients and perceived patient satisfaction

• Outcomes of the health check and follow-up

• Collaboration with other primary care professionals during the health check

• Practical and logistical aspects of the health check

The principal investigator (MG) moderated all focus groups. An assistant moderator was present during each meeting to take field notes and to ask for a clarification of comments when this was considered necessary. The meetings were both audio- and video-recorded. These recordings were transcribed verbatim. The transcripts were summarized, and these summaries were sent to the participants for their critical approval. All participants approved with the summaries, with a few added clarifications.

### Patients’ experiences

We explored the experiences of the patients with the use of a short written questionnaire that was sent by post to all responders of the initial screening questionnaire. This group included patients who had gone through the entire screening programme, as well as those patients who had only returned the initial screening questionnaire and were not diagnosed with an elevated risk and therefore did not participate in the additional screening steps. The questionnaire was sent on behalf of their GP with an accompanying letter that explained about the purpose of the research project. The questionnaire contained two open-ended questions: we asked participants to name one aspect of the health check that they had appreciated, and one that needed improvement. By using open-ended questions we were hoping to get a broad palette of patients’ thoughts and ideas, without pre-structuring their minds.

### Data analysis

As we wanted to let themes emerge from our data instead of starting our analysis from a predefined model we used a thematic content analysis based on grounded theory principles [15]. In grounded theory themes and constructs are formed and interpreted through a process of constant comparative analysis [16].

Based on the summaries of the focus groups three researchers (MG, MS and GJD) independently identified the main themes of the discussions. The transcripts were coded accordingly by MG using the software programme Atlas.ti®. In consecutive rounds of discussion main- and sub-themes were further specified by the researchers.

The answers from the returned patient questionnaires were imported into a database file. This database file was used by the same three researchers (MG, MS and GJD) to again identify important themes. Initially this was done manually by all three researchers. Through several rounds of discussion amongst the researchers (in which the identified themes were compared and discussed) the themes were further elaborated and specified. Eventually a second set of main- and sub-themes was identified.

In a final round of discussion between MG, MS and GJD the themes that had emerged from the focus groups and the patient questionnaires were merged into one single model and illustrative quotations were identified by MG. The quotations were translated from Dutch into English. In some cases quotes were slightly edited after translation to enhance reading clarity.

## Results

All caregiver focus groups were conducted between October and December 2009. All involved caregivers participated, with the exception of four medical receptionists, who were unable to be present for various practical reasons (working part-time, being ill, not working at the centre anymore). The first focus group consisted of three practice nurses. The second focus group consisted of five medical receptionists. The third focus group consisted of five GPs plus the manager of the healthcare centre. Each focus group met once, with the exception of the group of GPs, which met twice because some topics had not yet been exhaustively discussed by the end of the first meeting.

The patient questionnaires were sent by post in October 2009 to all responders of the original screening questionnaire (n = 1270). A total of 657 (52%) of these questionnaires were returned.

We will provide an overview of the different themes that emerged from the primary care professionals’ focus groups and patient questionnaires. Themes are accompanied by illustrative quotations (MR = medical receptionist, PN = practice nurse).

Generally, the themes discussed by patients were mainly related to the value of health checks and the procedures of the current health check, whereas the primary care professionals also reflected on the meaning and implications of offering health checks in primary care.

### Offering and receiving primary prevention

#### *Screening versus case-finding*

GPs were generally enthusiastic about offering the health check. It helped them to identify their high-risk population in a structured manner, where case-finding (the detection of high-risk patients during regular care consultations) often fell short of its purpose.

“Case-finding happens very ad hoc. For some people you do it much too often, and for others you never do it. So it’s a rather unorganized business. (GP2)

#### *Importance of primary prevention, offered by GPs*

Patients mostly appreciated being invited to participate in the health check. They valued primary prevention as important, and appreciated the fact that this programme was offered by their own GP.

“Abnormalities that otherwise might not have been discovered can now be discovered at an early stage” (Patient 2)

#### *Contents of the health check*

Even though most patients indicated that they were very happy to be able to participate in the health check, quite a number of patients would have wanted a more extensive health check. The additional tests that were most frequently mentioned by the patients were prostate testing and electrocardiography.

“What I remember is that it wasn’t very thorough. Only a questionnaire and blood testing. No ECG or anything likewise” (Patient 4)

“I had expected more of the health check. I can hardly believe that after such an examination, you have enough information to determine whether or not you have cardiovascular disease”. (Patient 5)

#### *Yield of the health check*

The practice nurses thought that the yield of the health check was high: many thus far unknown cases had been identified.

“What I really noticed was how many people with latent disease, or elevated blood glucose levels we have identified. And how many people with a well elevated LDL cholesterol” (PN1)

### Division of tasks

#### *Delegation of care*

During the health check, most care was delivered by medical receptionists and practice nurses instead of GPs. This idea of ‘delegation of care’ was received with enthusiasm by all primary care professionals. The medical receptionists discovered that they were well able to convey concepts such as the importance of managing blood pressure to patients.

“That you can explain things like: when diabetes runs in your family it’s important to be extra careful for cardiovascular disease. Or that a high blood-pressure is something that you don’t feel, so that is why we’re doing this health check”. (MR1)

The GPs perceived the training of medical receptionists as valuable. Some patients commented positively on the involvement of medical receptionists and practice nurses. Some others, however, felt that the receptionists were less knowledgeable and at times insecure.

“They made time for me, and everything was explained very clearly” (Patient 7)

“The examiner was insecure and indecisive. Please screen for knowledge and skills”. (Patient 8)

#### *Preparation and knowledge*

There was some disagreement about the amount of guidance and instructions that the medical receptionists and practice nurses needed. Where the GPs felt that there was a very clear and straightforward protocol, both the receptionists and practice nurses indicated that they would have benefitted from a more thorough preparation. In particular, the practice nurses indicated that they did not only want to know **what** had to be done (operational knowledge), but that they also wanted to have the background knowledge (medical knowledge) to help them understand **why** things were done in a specific way, so they could better inform their patients.

“Well, eventually we received a flow-chart that explained how to deal with the lab results. When to refer a patient. And I think that could have been discussed with us in greater detail. (…) Because you get this piece of paper, and that’s all you get”. (PN2) “I would have liked to be able to explain to patients things like: how serious is it to have slightly elevated urine albumin levels? (…) That was completely new subject matter to me”. (PN3)

The medical receptionists that were not directly involved in the health check by examining patients were confronted with patients’ questions about the programme at the reception desk or by phone. They felt unprepared to answer these questions.

“People would keep calling for their results. They thought that we would be able to give them the results”. (MR2) “But I was unable to answer their questions. Often you couldn’t answer their questions” (MR3)

Patients who were diagnosed with an elevated risk were referred to their GP for further treatment. Some GPs felt suddenly confronted with a patient where they would otherwise not have considered treatment, but now the results of the health check indicated that a patient did actually need treatment. This made them feel challenged in their judgement skills in everyday practice.

“One of the things that was difficult for me, was that sometimes I had to re-check the guidelines (…). Because sometimes I was told that I had to do something with a specific patient outcome (…). And I would say: ‘do I really have to?’. ‘Yes, you have to prescribe medication for that’. So I took out all the risk charts, because I thought I didn’t have to. Because he didn’t smoke and didn’t have a high blood-pressure. So well, that was the most difficult aspect for me” (GP4)

### Approaching the participants

#### *Dealing with healthy patients*

GPs discussed the most appropriate way to invite patients. The invitation letter was designed to result in a high participation rate: the dangers of cardiovascular disease and the importance of early detection were stressed explicitly. Some GPs indicated that they would opt for a less aggressive approach in future health checks. The health check was targeting healthy individuals without any physical complaints, and therefore there was no reason to cause any unnecessary worries.

“I think that we, in primary care, shouldn’t exaggerate when we invite individuals of 40 to 75 years of age in such an open manner. But that’s my personal opinion about healthcare. It’s lifestyle that we speak about, and potential risks”. (GP5)

“And you have every reason to approach this group in a very reassuring manner: that there’s a small group that will turn out to have an elevated risk down the end of the line” (GP2)

#### *Communicating the results, avoiding unnecessary harm*

A second letter, to invite patients with a possibly elevated risk (based on their answers to the screening questionnaire and their blood tests) for a follow-up consultation at the practice, was strongly emphasizing this possibly elevated risk. The letter did not contain any further information about why the risk was elevated. In some patients this resulted in an upset reaction and the request for an urgent consultation with their GP. In most of the cases the elevated “risk” was rather innocent: e.g. a slightly elevated blood glucose or cholesterol level. The GPs and practice nurses felt that these patients were unnecessarily harmed by this approach.

“I was quite scared by the invitation letter because it stated that I had an elevated risk for cardiovascular disease”. (Patient 9)

“The letter that I received after the examinations was quite terrifying, while in the end it turned out that hardly anything was wrong”. (Patient 10)

Patients indicated that they would have preferred to receive more detailed information about their results. They also stressed the importance of an easily accessible service point for additional information and questions.

“I would have liked to receive a written report of the lab results. What are the results, and what do they mean?” (Patient 11)

“I had to phone several times in order to receive my results” (Patient 12)

#### *Providing room for questions*

Patients that did not have an elevated risk were not invited for a follow-up consultation, but only received a letter stating that their results were normal. Also these patients indicated that they would have liked more detailed information about their results and the opportunity to ask questions.

“I didn’t get anything to take home. Nothing that I can use to compare in future examinations: cholesterol levels, blood pressure, other blood results, weight” (Patient 14)

“I would have liked a short evaluation of the results with my GP” (Patient 16)

The practice nurses realized that they did not have any information about how this group of patients felt about the health check.

“Well, I wonder how people that only received a letter informing them that they had a low risk (…) those that were not invited for a second consultation, how they experienced that. We don’t have any information on that. But I do wonder. Whether they feel a bit discarded, like: ‘Well, I had my bloods taken, and apparently everything was all right, but really… I don’t know anything’. Because what I did hear (…) is that some people phoned because they wanted their lab results anyway, or that some still had unanswered questions”. (PN3)

### Follow-up after the health check

#### *Importance of follow up and continuing care*

Because of all the effort that was invested into developing the health check, there had been less attention for the development of follow-up programmes. Patients with a clearly elevated risk or manifest disease were referred to their GPs. But there was also a group of patients that did have one or more risk factors, but did not exceed the threshold for referral to their GP. And even though there were plans for a lifestyle and risk management programme in the near future, there was no clear policy about how to follow-up on these patients.

“There were also people that would say: so what’s next? Because there was no follow-up here. We knew, of course, that in the future we will have a cardiovascular risk management programme. So I have informed patients of that, like: ‘By the end of this year or in the beginning of next year, there will be a programme that you will be invited for, considering the conditions that you currently meet’”. (PN1)

Some patients were disappointed as well about this lack of follow-up after receiving a one-time lifestyle advice.

“I was disappointed that I wasn’t offered any coaching in order to change my lifestyle. When you’ve lived in the same way for 62 years intensive coaching is required to achieve a radical change in this”. (Patient 17)

Both GPs and practice nurses realized during our evaluation that even though there were plans for follow-up after this health check, no arrangements regarding this follow-up had been made. There was no system for tracking down the different groups of patients in order to assign them to an appropriate follow-up program.

“And do you think that you’ll be able to track down these people by then?” (Moderator)

“I hope so…” (PN1)

#### *Integration of the health check with everyday clinical practice*

The GPs also realized that even though a lot of information regarding family history and lifestyle had been collected during the health check, most of this information was not readily available for them during their regular consultations because a separate computer database had been used for the health check.

“I thought it was shame, and I don’t know why it happened… Many things were registered during the health check. I have seen a number of patients that had participated in the health check during my consultations. But I had to ask them all over again whether they smoked or they didn’t. (…) And that wasn’t registered”. (GP2)

## Discussion and conclusions

### Summary of main findings

In this study we evaluated the most important issues that arose during the implementation of a cardiometabolic health check in primary care. We found that GPs were enthusiastic about offering a health check. They preferred systematic screening over case-finding, both in terms of yield and workload. The level of patient participation was high and most participants were enthusiastic about the health check being offered by their GP. Despite their enthusiasm, the GPs realized that they lacked experience in the design and implementation of a structured, large-scale prevention programme. This resulted in suboptimal instruction of the involved practice nurses and medical receptionists, an aggressive recruitment strategy and shortcomings in communicating the outcomes of the health check as well as in the provided follow-up programmes.

Based on our findings we have created a checklist (Table 1) with potential issues that can rise during the implementation of a health check in primary care. Each item is accompanied by a number of probing questions that designers of such a programme can reflect upon.

**Table 1:** Checklist for designers of a health check

	
**Involvement of various primary care professionals**	*Which primary care professionals are going to be responsible for the different aspects of the health check? How have the different groups of primary care professionals (e.g. doctors, nurses, receptionists) been involved in the planning phase?*
	*Is there a shared sense of mission and ownership?*
**Operational knowledge and responsibilities**	*Are all primary care professionals aware of their role in the programme?*
	*Are the responsibilities clearly defined?*
	*Is there some kind of coordination?*
	*Who will be able to answer primary care professionals’ questions regarding the programme, both before and during the programme?*
	*Will there be regular evaluation meetings?*
**Scientific effectiveness**	*Is there any scientific evidence regarding the effectiveness of the screening programme available?*
	*What are the expected costs and benefits?*
	*What are possible limitations of the programme?*
	*Is there any risk of causing physical of psychological harm in participating patients?*
**Biomedical knowledge**	*Does everyone feel well-prepared and capable for their role in the programme?*
	*Is there a need for additional biomedical knowledge?*
	*Is everyone up-to-date with the latest relevant medical guidelines?*
**Approach of healthy individuals**	*How will participants be approached?*
	*Is the approach expected to result in a high participation rate?*
	*Will the possible benefits and harms of the health check be clearly communicated to the participants beforehand?*
	*How will we make sure not to cause any unnecessary worries or harm?*
	*Are there any financial costs for participating patients?*
**Communicating the results and providing room for questions**	*How will the results be communicated to patients?*
	*How will we make sure not to cause any unnecessary worries, due to wrong interpretations of the results?*
	*Who can patients turn to in case they have questions?*
	*Is this “helpdesk” easily approachable?*
	*How will patients with a low-risk be approached?*
	*Will they too receive a copy of their results?*
**Integration with everyday practice**	*How will the health check be integrated in routine medical practice?*
	*Is the office staff expected to perceive an additional administrative burden because of the screening programme?*
	*How will they be assisted in this?*
	*Will the results of the health check be readily available in the electronic medical records?*
**Follow-up programmes**	*Which follow-up programmes will be offered to detected cases?*
	*How many cases are anticipated to be detected during the health check?*
	*Will it be possible to identify these detected cases at a later stage?*
	*What are the criteria that need to be met in order to qualify for a follow-up programme?*
	*Will there be anything that can be offered to patients that do not qualify for the predetermined follow-up programmes?*

### Comparison with existing literature

GPs have considered prevention as a fundamental part of good clinical practice for decades [17,18]. The increasing pressure of chronic diseases on the health care system, as well as social and technical changes [19] urge GPs to develop new models of working in order to be able to cope with increasing demands [20]. These new models challenge the classic primary care paradigm of demand-driven, instantaneous care, to evolve into a more anticipatory, service-oriented and continuing kind of care. In our current research the GPs indicated that they felt confronted with a new workflow that required a different patient approach and challenged them in their conventional modes of operation. They had not anticipated the large demands that the health check would cause and were unable to provide follow-up to all participants.

Some patients indicated that they found the way they were approached rather provocative and distressful. This is in line with warnings about the psychological harm that health checks can cause [21], although there is less evidence about longer-term negative effects [22]. Literature has shown that patients are not very well able to judge and interpret their own risks [23,24] and may have unrealistic expectations about the value of diagnostic tests [25]. This implies that patients need assistance in translating their test results into understandable concepts to effectively manage their risks. Once risk-factors have been identified, it would be unethical to leave the worried patient to himself. Especially because the initial health check was not offered at the patients’ but at the physicians’ initiative.

### Strengths and limitations

The experiences described in our research paper were gathered during the evaluation of a cardiometabolic health check in one primary healthcare centre in an urbanized setting. We do not pretend to provide and exhaustive account of all factors that can possibly be involved during the implementation of such programmes. Although the absolute numbers were small and some circumstances were quite specific to the research setting, we do believe that our qualitative data provide some very relevant and credible insights into the general implementation of prevention programmes in primary care. We therefore believe these results can also be of great value to others, especially because – up to our knowledge – no other articles about these specific issues have been published.

### Implications for future research or clinical practice

Our research indicates that a number of fundamental issues may arise when GPs organize a systematic screening programme in their practice. These issues are related to the preparation of all involved staff, the importance of integration with everyday clinical practice, the approach of patients and communicating their results and the provision of adequate follow-up programmes. Future screening programmes can take into account the identified challenges. Thorough process evaluations of such programmes can lead to a refinement of our recommendations.

### Declarations

#### *Ethical approval*

Ethical approval for this study was granted by the Medical Ethics Committee of Maastricht University Medical Centre (MEC 09-4-054).

## Abbreviations

CMD: Cardiometabolic diseases; ECG: Electocardiography; GP: General practitioner; MR: Medical receptionist; PN: Practice nurse; PSA: Prostate-specific antigen.

## Competing interests

The authors declare that they have no competing interests.

## Authors’ contributions

MG was the main researcher, and was involved in the conception and design of the study, data collection and analysis and was the main author of the various drafts of the manuscript. MS and GJD made significant contributions to the conception and design of the study, the data analysis, and critically commented on various drafts of the manuscript. JAK made significant contributions to the conception and design of the study and critically commented on the final draft of the manuscript. WvdM and GJ were involved as local coordinators of the primary prevention programme in the primary healthcare centre. They were both part of a focus group, critically commented on the summary of the focus groups and also critically commented on the final manuscript. All authors read and approved the final manuscript.

## Pre-publication history

The pre-publication history for this paper can be accessed here:

http://www.biomedcentral.com/1471-2296/15/132/prepub
